# Downshifting Yeast Dominance: Cell Physiology and Phospholipid Composition Are Altered With Establishment of the [*GAR*^+^] Prion in *Saccharomyces cerevisiae*

**DOI:** 10.3389/fmicb.2020.02011

**Published:** 2020-08-25

**Authors:** Gordon A. Walker, Clark M. Henderson, Peter Luong, David E. Block, Linda F. Bisson

**Affiliations:** Department of Viticulture and Enology, University of California, Davis, Davis, CA, United States

**Keywords:** *Saccharomyces cereivisiae*, [*GAR*^+^], prion, lipidomics, oxygen consumption, fermentation

## Abstract

Establishment of the [*GAR*^+^] prion in *Saccharomyces cerevisiae* reduces both transcriptional expression of the *HXT3* hexose transporter gene and fermentation capacity in high sugar conditions. We evaluated the impact of deletion of the *HXT3* gene on the expression of [*GAR*^+^] prion phenotype in a vineyard isolate, UCD932, and found that changes in fermentation capacity were observable even with complete loss of the Hxt3 transporter, suggesting other cellular functions affecting fermentation rate may be impacted in [*GAR*^+^] strains. In a comparison of isogenic [*GAR*^+^] and [*gar*^–^] strains, localization of the Pma1 plasma membrane ATPase showed differences in distribution within the membrane. In addition, plasma membrane lipid composition varied between the two cell types. Oxygen uptake was decreased in prion induced cells suggesting membrane changes affect plasma membrane functionality beyond glucose transport. Thus, multiple cell surface properties are altered upon induction of the [*GAR*^+^] prion in addition to changes in expression of the *HXT3* gene. We propose a model wherein [*GAR*^+^] prion establishment within a yeast population is associated with modulation of plasma membrane functionality, fermentation capacity, niche dominance, and cell physiology to facilitate growth and mitigate cytotoxicity under certain environmental conditions. Down-regulation of expression of the *HXT3* hexose transporter gene is only one component of a suite of physiological differences. Our data show the [*GAR*^+^] prion state is accompanied by multiple changes in the yeast cell surface that prioritize population survivability over maximizing metabolic capacity and enable progeny to establish an alternative adaptive state while maintaining reversibility.

## Introduction

*Saccharomyces cerevisiae* is a remarkably adaptable organism specialized in dominating fermentative environments (Karpel et al., 2008; Bisson and Karpel, 2010). Strains used in the commercial production of food and beverages have been selected for particular genetic adaptations that facilitate their production proficiency (Dashko et al., 2014). Recent research has revealed that yeast contain a global network of regulatory proteins, which facilitate dynamic responses to various types of environmental stress in a manner that is transmitted automatically to progeny cells (Gasch, 2007; Bisson et al., 2016; Brion et al., 2016). Proto-prion proteins contain intrinsically disordered domains that can spontaneously refold into the [*PRION*^+^] form in response to loss of cellular homeostasis (Garcia and Jarosz, 2014; Chakrabortee et al., 2016). Refolding of these domains in regulatory proteins constitutes a type of structural “switch” which leads to sequestration or differential associations, and can even confer novel functions to the target protein (Harvey et al., 2017). These epigenetic “switch” elements display characteristics of non-Mendelian inheritance.

There is mounting evidence that the majority of yeast prions are not vestigial disease states (Shorter and Lindquist, 2005; Halfmann and Lindquist, 2010; Wickner et al., 2011; Liebman and Chernoff, 2012). Instead, yeast prions represent a common mechanism for generating transiently heritable phenotypic diversity that can confer certain advantages under specific detrimental environmental conditions (Halfmann et al., 2012; Byers and Jarosz, 2014; Chernova et al., 2014; Jarosz et al., 2014b; Chakrabortee et al., 2016). It has been hypothesized that these prion states offer a population dynamic survival strategies without permanently affecting the fitness of the population (Shorter and Lindquist, 2005). Yeast prions are dynamically propagated generation to generation through the action of molecular chaperones that belong to the family of Heat Shock Proteins (HSPs) (Masison and Reidy, 2015). HSPs are activated by the cumulative effect of the Adaptive Stress Response Networks (ASRNs), which respond dynamically to different types of environmental stress (Bisson et al., 2016).

The prion known as, [*GAR*^+^] (Resistant to Glucose-Associated Repression), allows yeast to circumvent glucose-repression of alternative carbon sources and utilize alternative carbon substrates in the presence of glucose (Brown and Lindquist, 2009). The [*GAR*^+^] prion is non-amyloid in nature, similar to other recently identified non-amyloid epigenetic elements that meet the genetic criteria for yeast prions (Brown and Lindquist, 2009; Chakrabortee et al., 2016). In a laboratory context, [*GAR*^+^] cells can be identified or induced with a selective media, glycerol glucosamine medium (GGM). Growth on this medium indicates the ability to bypass glucose-repression, which is enabled by a mutation or induction of the [*GAR*^+^] prion. In a natural context [*GAR*^+^] is induced by the presence of specific bacteria and bacterially produced organic acids (Jarosz et al., 2014a; Garcia et al., 2016; Ramakrishnan et al., 2016). The [*GAR*^+^] prion is stable over 100x generations on non-selective media (Jarosz et al., 2014a). The prion is occurs at a particular frequency for each strain, depending on ecological background (Jarosz et al., 2014b). The inherent frequency of [*GAR*^+^] can be determined by “curing” a strain of [*GAR*^+^] by expressing a dominant-negative copy of Hsp70, then plating the “cured” strain on GGM and assessing frequency of the [*GAR*^+^] phenotype (Garcia et al., 2016; Walker et al., 2016).

Mechanistically, establishment of the [*GAR*^+^] prion appears to be dependent on a structural change in the plasma membrane ATPase, Pma1. Refolding of Pma1 by a yeast HSP70, Ssa1, results in a shift of primary association of Pma1 from Mth1 to Std1 (Brown and Lindquist, 2009). Mth1 and Std1 are important co-repressors of hexose transporter gene expression (Kim et al., 2006). The bound forms of these transcriptional factors are resistant to normal modes of protein degradation (Kim et al., 2006) and protection of a regulatory element by complexing with a stable plasma membrane protein may impact regulatory activity within the cell. Establishment of [*GAR*^+^] results in a ∼40x decrease in hexose transporter 3 (*HXT3*) mRNA transcript levels (Brown and Lindquist, 2009). However, this analysis was done in laboratory strains growing under laboratory conditions and at the point of diauxic shift.

Decreased *HXT3* expression is significant because Hxt3 is a ubiquitous high capacity hexose transporter that is important for fermentation efficacy and alcohol tolerance (Karpel et al., 2008). Under typical native fermentation conditions, ethanol accumulates in the absence of molecular oxygen and prevents diauxie from occurring. Therefore we were interested in assessing phenotypic expression of the [*GAR*^+^] prion under native fermentative conditions in a native vineyard isolate.

Simple loss of the *HXT3* gene (*hxt3Δ*) does not enable growth on the prion-selective medium (Brown and Lindquist, 2009), thus loss of the gene alone does not allow cells to utilize alternative carbon sources in the presence of glucose. Cells harboring [*GAR*^+^] appear to be less competitive during fermentation with other microbes present in the media, allowing for increased bacterial abundance (Walker et al., 2016). A population can utilize the prion in a mixed microbial environment to diversify carbon source utilization and increase chances of survival (Jarosz et al., 2014a). The fact that wine spoilage bacteria can induce this prion suggests that proton stress may play a role in [*GAR*^+^] induction (Jarosz et al., 2014a; Ramakrishnan et al., 2016; Walker et al., 2016). We hypothesize that the establishment of the [*GAR*^+^] prion is an adaptation to stressful mixed microbial environments retooling cellular metabolism and plasma membrane functionality to enable the yeast population to employ an alternative metabolic survival strategy.

Fermentations comparing WT [*gar*^–^] and [*GAR*^+^] cells have demonstrated that the presence of the prion leads to a reduction in “fermentation capacity,” which is defined by the ability to efficiently deplete nutrients, dominate microbial competition, and complete the conversion of hexoses into ethanol (Jarosz et al., 2014b; Walker et al., 2016). Fermentation capacity is further determined by a combination of the alcohol tolerance of individual cells and metabolic flux across the population (Henderson and Block, 2014). Numerous physiological factors impact ethanol tolerance and fermentation capacity.

Previous work has shown the correlation of plasma membrane lipid composition with fermentation progression, kinetics, and ethanol tolerance (Henderson et al., 2013a; Henderson and Block, 2014). Isogenic [*gar*^–^] and [*GAR*^+^] cells were evaluated for differences in a variety of properties associated with plasma membrane functionality: morphology, integral protein distribution, changes in phospholipid composition, and oxygen uptake kinetics.

## Materials and Methods

### Yeast Strains Used

The yeast strains used were UCD932 (BA2), which was isolated from a vineyard in Italy (Mortimer et al., 1994), and UCD932 *hxt3Δ*, which contains a deletion of the *HXT3* gene (Karpel et al., 2008). UCD932 Hxt3-GFP [*gar*^–^] was obtained from Karpel et al. (2008) and a [*GAR*^+^] derivative carrying the GFP fusion was isolated as described below. Strain UCCT 1405 Pma1:m:Neon:KanMX was a gift from Dr. Kiersten A. Henderson of the Gottschling Laboratory at Fred Hutchinson Cancer Center.

### Fermentations With Isogenic [*gar*^–^] and [*GAR*^+^] Strains of UCD932 and UCD932 *hxt3Δ*

The [*GAR*^+^] prion was induced in these strains (and all following strains) by plating on to GGM selective media (Brown and Lindquist, 2009) then restreaking on GGM for two successive colony generations. Colonies were then restreaked onto permissive 2% Yeast Peptone Dextrose agar plates (YPD) [1% Bacto™ Yeast Extract (Becton, Dickinson and Company, Sparks, MD, United States), 2% Bacto™ Peptone (Becton, Dickinson and Company, Sparks, MD, United States), 2% Dextrose (Amresco^®^, Solon, OH, United States)] for one generation. The presence of the prion was confirmed by assessment of the [*GAR*^+^] phenotype as described in Ramakrishnan et al. (2016).

Single colonies each of UCD932 [*gar*^–^] and [*GAR*^+^] and UCD932-1 *hxt3Δ* [*gar*^–^] and [*GAR*^+^] were picked from YPD plates and inoculated into 10 mL of synthetic juice or Minimal Must Media (MMM) with 208 mg/L Yeast Nitrogen Equivalents (YNE) and grown on a rotary drum at 25°C for 48 h (Spiropoulos et al., 2000). The Absorbance (A_60__0 n__m_) of the cultures was determined, and 75 mL of MMM 208 YNE in 150 mL Erlenmeyer flasks was inoculated at 0.05 A_60__0 n__m_. Fermentations were performed with six biological replicates. Flasks were placed on an orbital shaker at 120 RPM at ∼25°C. Flasks were weighed daily to track fermentation progress via CO_2_ loss. The data presented is the mean and SD of the rate which was calculated by taking the derivative of the loss of CO_2_ (grams) per day.

### Evaluation of UCD932 [*gar*^–^] and [*GAR*^+^] Cell and Colony Morphology

Single colonies of UCD932 [*gar*^–^] and [*GAR*^+^] were inoculated into 5 mL of 2% YPD liquid media and allowed to grow overnight on a rotary drum at 25 °C. Cultures were then reinoculated at 0.1 A_60__0 n__m_ and allowed to reach mid-log phase (∼0.4 A_60__0 n__m_) before imaging. A wet mount of [*gar*^–^] and [*GAR*^+^] cells was prepared by placing 5 uL of the sample on a clean slide and adding a cover slip. Cells were captured using a 40X lens on a Celestron^®^ LCD Deluxe Digital Microscope (Celestron, Torrance, CA, United States). Population histograms, average cell size, and volume were assessed with the Scepter 2.0 Handheld Automated Cell Counter (EMD Millipore, Hayward, CA, United States). Cultures were diluted 1:10 into 1X Phosphate Buffer Saline (PBS) adjusted to 7.4 pH. The Scepter 2.0 was equipped with the 40 uM Sensors (EMD Millipore, Hayward, CA, United States). Histograms were downloaded from the Scepter Software version 2.1.

Single colonies of UCD932 [*gar*^–^] and [*GAR*^+^] were streaked onto Difco™ Wallerstein nutrient agar (WLN) (BD, Franklin Lakes, NJ, United States) to assess colony morphology. Colonies were photographed after 3 days of growth at 30°C. WLN medium contains bromocresol green as an indicator of pH and is used to differentiate acid-producing microbes from those that are not acid producing (Rodrigues et al., 2001).

### Fluorescence Microscopy and Staining of Yeast Strains

Single colonies of UCD932 [*gar*^–^] and [*GAR*^+^], UCD932 Hxt3-GFP [*gar*^–^] and [*GAR*^+^], and UCCT 1405 Pma1:m:Neon:KanMX [*gar*^–^] and [*GAR*^+^] were inoculated into 10 mL of YPD liquid and grown overnight on a rotary drum at 25 °C. Cultures were reinoculated and grown until mid-log phase/exponential phase (∼0.4A_60__0 n__m_). Stationary cells were taken from the original overnight cultures (∼1A_60__0 n__m_). Wet mounts were prepared and photographed with the Carl Zeiss AX10 Imager. A1 Microscope (Carl Zeiss, Oberkochen, Germany), Zeiss EX Plan-NEOFLUAR 100X oil immersion lens, with the Zeiss 38 HE Filterset (Ex:440/Em:550). Images were exported in the.tiff format using the Zeiss AxioVision acquisition software version 4.8 from Carl Zeiss. Similar exposure times were used for all images being compared.

### Assessing Fluorescence of UCD932 Hxt3-GFP

Single colonies of UCD932 Hxt3-GFP [*gar*^–^] and [*GAR*^+^] were picked and inoculated into 10 mL of 2% YPD Liquid and grown overnight on a rotary drum. Cultures were then reinoculated at 0.1 A_60__0 n__m_ and allowed to reach mid-log phase (∼0.4 A_60__0 n__m_) before imaging. Sample preparation was undertaken as advised by Amnis^®^ ImageStreamX^®^ MarkII Imaging Flow Cytometer (MilliporeSigma, Seattle, WA) instrument guidelines: ∼5×10^6^ cells/mL, washed and suspended in 1X PBS, and filtered through 70 μm mesh. 10,000 events were acquired with Amnis INSPIRE^®^ using the 488 nm laser and 60X magnification (Calvert et al., 2008). IDEAS^®^ software was used for data analysis. The yeast population was gated to capture in focus single cells, budding cells, and double cells. GFP expression was quantified based on the mean fluorescence intensity of > 2000 individual events counted per population (single cell, budding cell, double cell). Since only one fluorophore was being assessed and brightfield images were not used for additional analysis no compensation was applied in this sample data.

### Culture Preparation for Phospholipid Analysis

Single colonies of UCD932 [*gar*^–^] and [*GAR*^+^] were selected from 2% YPD agar plates and inoculated into 10 mL MMM prepared at 208 mg/L YNE and allowed to grow on a rotary drum at 25°C for 48 h. These cultures were then used to inoculate 150 mL of MMM 208 mg/L YNE in 250 mL baffled flasks at 0.05 A_66__0 n__m_. Fermentations were performed in triplicate. Flasks were weighed daily to track fermentation kinetics through the evolution of CO_2_. On day 2 (early) and 7 (late) of fermentation, 50 mL samples were taken, spun down at 8000 × g for 8 min discarding the supernatant, washed in 50 mL of sterile H_2_O, and spun down again removing the supernatant. Cells were then resuspended in 10 mL of sterile DI H_2_O, transferred to 15 mL conical centrifuge tubes (Fisher Scientific, Pittsburgh, PA, United States), spun down 10 min at 4000 × g, supernatant aspirated and pellets frozen in liquid nitrogen then stored at −80°C.

### Lipid Extraction and Preparation and HPLC-MS Analysis of Phospholipids and Sterols

Briefly, lipids were extracted from frozen and thawed yeast pellets using a modified Bligh-Dyer method that included a 5% formic acid extraction step to improve recovery of acidic phospholipids (Henderson et al., 2011). Phospholipids were quantified using normal phase high-performance liquid chromatography coupled on-line to electrospray ionization-tandem mass spectrometry operating in negative ion detection mode (Henderson et al., 2011). Sterols were quantified by flow injection analysis using atmospheric pressure chemical ionization-mass spectrometry (Henderson et al., 2013b). Instrument data files were converted to mzXML format for analysis utilizing MZmine version 2.1 (Pluskal et al., 2010). All lipid analytes were normalized to surrogate internal standards and quantified using spiked calibration curves (Henderson et al., 2013a).

### Phospholipid Data Analysis

The data was formatted and exported in a.csv file format and uploaded onto the MetaboAnalyst online platform (Xia et al., 2015). Partial least squared discriminant analysis (PLS-DA) was used to visualize the relative differences between significant phospholipid species present in [*gar*^–^] vs. [*GAR*^+^] fermentations, and day 2 vs. day 7 fermentation phospholipid species. The significance of phospholipid species was determined by pair-wise *t*-test and analysis of variance (ANOVA). All of the phospholipid species shown in the data were determined to be significant (*p* ≤ 0.05). For the lipidomics data presented in Figures 4, 5, an unprotected Fisher’s Least Significant Difference test was performed, letters were added to denote when values are significantly different from one another (*p* ≤ 0.05). Graphs and figures were generated using Prism 7 (GraphPad.com).

### Tracking of Dissolved Oxygen During Fermentation

Single UCD932 [*gar*^–^] and [*GAR*^+^] colonies were inoculated into 10 mL of sterile filtered Chardonnay juice and placed on a rotary drum to grow at 25°C for 48 h. 250 mL Erlenmeyer flasks were fitted with PreSens PSt3 Oxygen Sensor Dots (PreSens, Regensburg, Germany) and autoclaved. Flasks were aseptically filled with 150 mL of sterile filtered Chardonnay, allowed to warm to room temperature, and inoculated at 0.05 A_60__0 n__m_ with the UCD932 [*gar*^–^] or [*GAR*^+^] cultures. Fermentations were performed in triplicate. The PreSens Fibox 3 LCD Trace fiber optic oxygen transmitter was used in conjunction with the PS3t dots to track dissolved oxygen over the first 16 h of fermentation. A pair-wise *t*-test was performed to determine when the Brix between conditions was significantly different (*p* ≤ 0.05), asterisks have been added to Figure 6 to denote significance.

## Results

### Loss of Hxt3 Alone Is Not Sufficient to Explain the Fermentation Defects Observed in Wine Strains Harboring [*GAR*^+^]

Cells harboring [*GAR*^+^] generally show a decrease in fermentation capacity, especially when challenged with microbial competition (Walker et al., 2016). The ∼40X decrease in expression of the important *HXT3* transporter in [*GAR*^+^] cells (Brown and Lindquist, 2009) could explain the slower kinetics of fermentation. However, this analysis was done using laboratory strains at the point of entry into diauxie, a state not typically found during natural juice fermentations.

**FIGURE 1:**
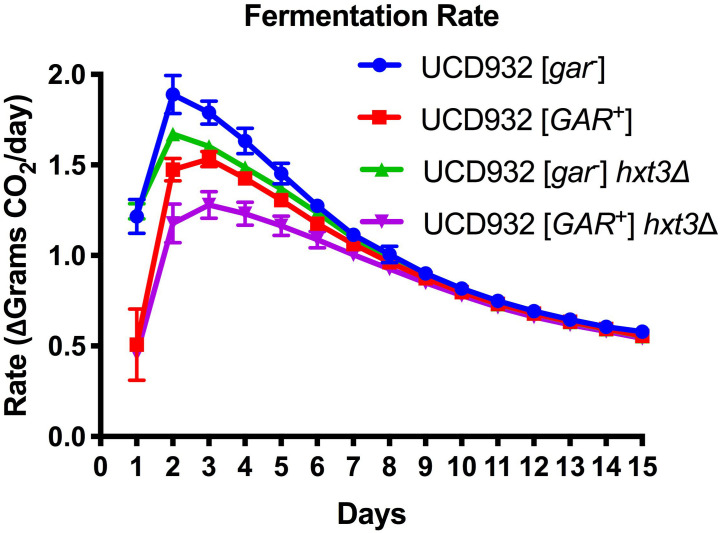
Fermentation Rate is Affected by [*GAR*^+^] Independently of Loss of *HXT3.* Data in this figure are the mean and standard deviation of the derivative of the rate of CO_2_ loss (in grams) per day of six biological replicates for each condition presented. This graph shows the rate of fermentation for all strains is compared together in synthetic juice (MMM). The asterisks denote significantly different values between conditions as determined by paired sample *t*-test (*p* ≤ 0.05). Error bars represent standard error of the mean.

To test the hypothesis that loss of *HXT3* is responsible for this decrease in fermentation rate in [*GAR*^+^] wine yeast cells, fermentations with a UCD932 *hxt3Δ* null mutant were performed. Induction of the prion resulted in a decrease in fermentation rate (Figure 1) as previously reported (Walker et al., 2016). Deletion of *HXT3* in the wild type strain likewise led to a decrease in fermentation rate (Figure 1), also consistent with previously published data on the impact of loss of *HXT3* (Karpel et al., 2008). If loss of *HXT3* alone explained the fermentation defect of [*GAR*^+^], the fermentation rate of UCD932 *hxt3Δ* [*gar*^–^] would be predicted to be indistinguishable from that of UCD932 *hxt3Δ* [*GAR*^+^]. However, induction of the [*GAR*^+^] prion in an *hxt3* deletion strain led to a further decrease in fermentation rate. Thus, in the absence of functional Hxt3, the only known transcriptional target of the [*GAR*^+^] prion, fermentation capacity decreases further upon induction of the prion, suggesting that there are other factors affecting the fermentation capacity of [*GAR*^+^] cells.

### UCD932 [*GAR*^+^] Cells and Colonies Display Morphological Differences

**FIGURE 2:**
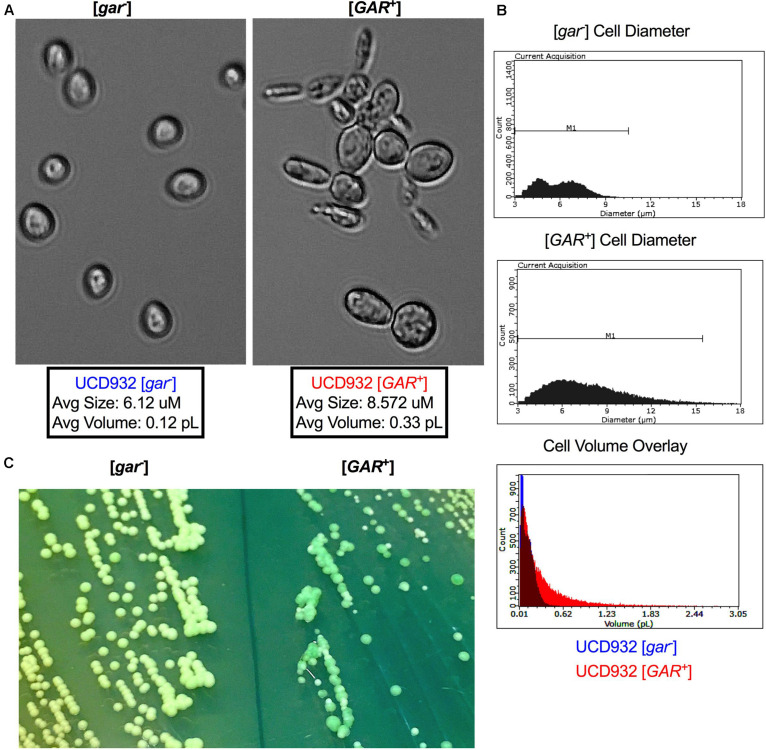
Cell and Colony Morphology of UCD932 [*gar*^–^] and [*GAR*^+^]. **(A)** UCD932 [*gar*^–^] and [*GAR*^+^] cells viewed with a 40X objective (Celestron) after growth into mid-log phase in YPD_liquid_. **(B)** Scepter 2.0 histogram of UCD932 [*gar*^–^] and [*GAR*^+^] cell diameter within a population also an overlay of cell volume for the above populations. **(C)** UCD932 [*gar*^–^] and [*GAR*^+^] cells streaked side by side onto the same WLN plate. The [*gar*^–^] colonies and media are distinctly lighter/whiter than the darker green more heterogeneous [*GAR*^+^] colonies. The inability of [*GAR*^+^] colonies to induce the same color change on WLN as [*gar*^–^] colonies suggests that the prion alters the ability to decrease the pH of the surrounding medium. Since glucose activates the proton pumping capacity of Pma1 (Shin and Loewen, 2011) this lack of medium acidification suggests that proton pump activation may be modified upon prion induction.

UCD932 [*GAR*^+^] cells can be differentiated from their [*gar*^–^] progenitors under the microscope (Figure 2). [*GAR*^+^] cells exhibit highly variable and often unusual cellular morphology, reminiscent of cells undergoing nutrient starvation or pseudohyphal growth (Figure 2A). Cells harboring [*GAR*^+^] are generally larger, and less uniform than WT [*gar*^–^] cells (Figure 2A). In addition to unusual morphology, [*GAR*^+^] cells display a propensity to clump together, forming dynamic cellular micro-aggregates (flocs). These changes in morphology are not observed when comparing *HXT3* wild-type cells to *hxt3* null strains (data not shown) thereby adding further support to the hypothesis that establishment of the prion has impacts other than the simple reduction of level of expression of the Hxt3 protein. [*GAR*^+^] cells exhibit a wider range of diameter and volume across the population as compared to [*gar*^–^] (Figure 2B).

In addition to differences in cellular size, induction of the prion also impacts colony morphology. On Wallerstein nutrient agar (WLN) plates, [*gar*^–^] colonies appear uniform and cream colored, and lighten the medium from blue to green indicative of a drop in pH of the surrounding media (Figure 2C). [*GAR*^+^] colonies are variable in size and color and do not show the same change in color of the medium (Figure 2C). The larger [*GAR*^+^] colonies on the WLN plate appear distinctly greenish and darker while the smaller colonies are lighter (Figure 2C). UCD932 [*GAR*^+^] colonies are often more segmented, a darker shade of green, and acidify the surrounding agar more slowly. This suggests that altered membrane functionality in [*GAR*^+^] cells results in differences in uptake or absorption of the dye present in the plate and the reduction in medium acidification could potentially be due to changes in Pma1 activity. Additionally, the colonies on the [*GAR*^+^] plate are not uniform in size and color, indicative of heterogeneity within the population. Replating of different colony types or sectors yielded the same variety of colony morphology even with repeated restreaking, and population assessments via comparative tip-plating on both GGM and YPD indicated that populations arising from the different colony types and sectors maintained presence of the prion and the ability to fully grow on the selective GGM medium (data not shown). The prion appears to lead to greater cell surface population diversity, suggesting that the prion enables cells within the population to express a variety of growth phenotypes.

### Fluorescently Labeled Integral Plasma Membrane Proteins, Pma1 and Hxt3, Display Differential Distribution in [*gar*^–^] vs. [*GAR*^+^] Cells

**FIGURE 3:**
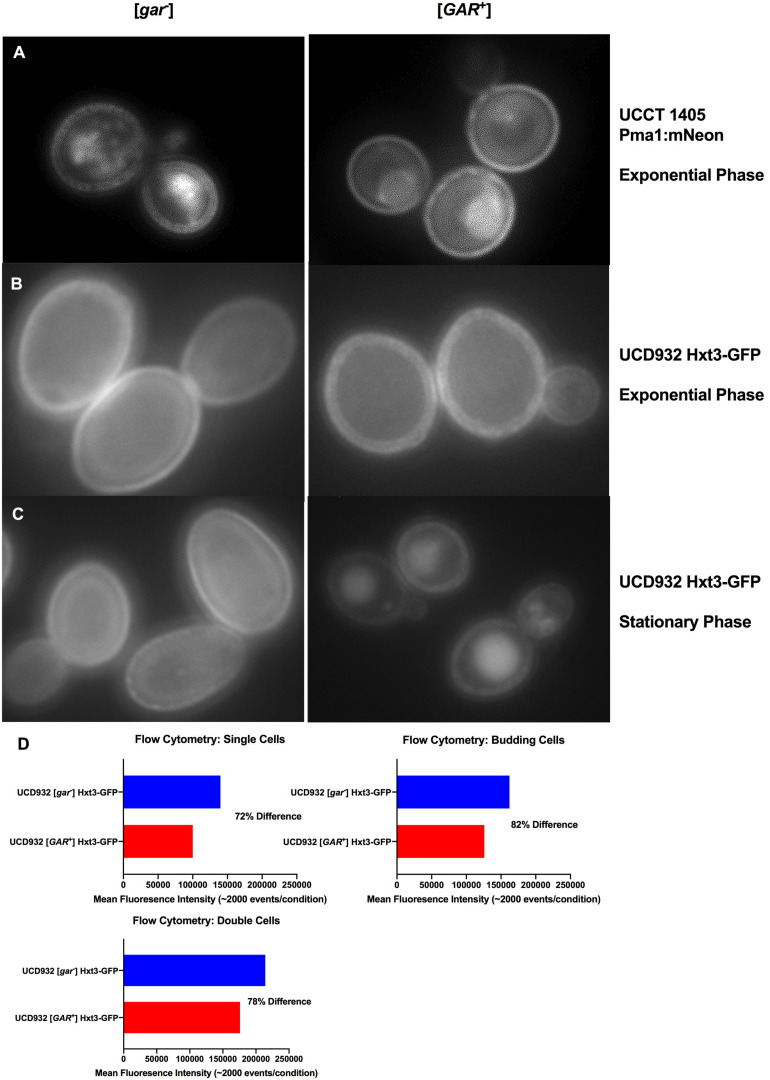
Localization of Integral Membrane Proteins Hxt3 and Pma1.**(A)** Pma1:mNeon [*gar*^–^] and [*GAR*^+^] in exponential phase at 100x with 1s exposure. **(B)** UCD932 Hxt3-GFP [*gar*^–^] and [*GAR*^+^] in exponential phase at 100x with 1s exposure. **(C)** UCD932 Hxt3-GFP [*gar*^–^] and [*GAR*^+^] in stationary phase at 100x with 1s exposure. Also see supplemental information (Supplementary Figures S3A–C) for multiple alternative fields of view for **(A–C)**. **(D)** Mean fluorescence of exponential phase UCD932 Hxt3-GFP [*gar*^–^] vs. [*GAR*^+^] for in focus single cells, budding cells and double cells. Each sub-population (single, budding, and double cell) represents the mean intensity of >2000 events as quantified by the ImagestreamX Mark II flow cytometer.

Yeast membranes are highly dynamic composites with functional integral proteins distributed non-uniformly, and organized into microdomains (Bagnat et al., 2000; Merzendorfer and Heinisch, 2013). A major component of these microdomains is the plasma membrane ATPase, Pma1p, which makes up ∼50% of the integral membrane protein in the cell (Ambesi et al., 2000). Given the importance of Pma1 in maintaining intracellular homeostasis, its organization and loading into the membrane have major implications for cellular function (Morsomme et al., 2000). To better understand the dynamics of [*GAR*^+^] membrane organization, UCCT1405, a strain with a functional Pma1:mNeon fusion was compared between [*gar*^–^] and [*GAR*^+^] states (Figure 3A). Generally, UCCT1405 [*gar*^–^] cells displayed typical distinct punctates of the mNeon moiety, consistent with previous observations of this protein in wild type cells. The [*GAR*^+^] UCCT1405 cells display more diffuse fluorescence across the cell membrane (Supplementary Figure 3A). This observation suggests that localization of Pma1 into microdomains may be different for [*GAR*^+^] cells (Ferreira et al., 2001; Malinska et al., 2003).

*HXT3* transcripts were reported to drop ∼40X in cells harboring [*GAR*^+^] (Brown and Lindquist, 2009). We evaluated Hxt3 protein levels directly in the plasma membrane during the fermentation with a wine yeast. The distribution of Hxt3p was assessed during exponential growth on glucose in vineyard isolate UCD932 expressing a Hxt3-GFP fusion protein (Karpel et al., 2008). UCD932 [*GAR*^+^] exponential cells displayed differences in the membrane, appearing less uniform and more punctate (Figure 3B and Supplementary Figure 3B) as compared to the [*gar*^–^] control. Stationary phase cells of UCD932 [*gar*^–^] appeared similar to those taken from exponential phase. In contrast fluorescence appeared localized to internal structures indicative of degradation of Hxt3-GFP later in fermentation in the isogenic [*GAR*^+^] strain (Figure 3C).

Populations of exponential UCD932 [*gar*^–^] and [*GAR*^+^] strains containing the Hxt3-GFP fusion protein were assessed for fluorescence intensity by flow cytometry. Consistent with previous observations, the [*GAR*^+^] populations of single cells (∼2000 events), double cells and budded cells displayed roughly an 18–28% lower mean intensity of Hxt3-GFP fluorescence (Figure 3D.), indicative of down-regulation of expression of the *HXT3* gene as reported in Brown and Lindquist (2009). The average reduction in intensity across the entire population was 22.7% with a SD of ± 5%. Overall intensity of fluorescence appeared greater for the [*gar*^–^] UCD932 cells, implying higher levels of Hxt3 in the cell (Figures 3B–D).

### [*GAR*^+^] and [*gar*^–^] Cells Exhibit Differential Phospholipid Headgroup Composition Over the Course of Fermentation

**FIGURE 4:**
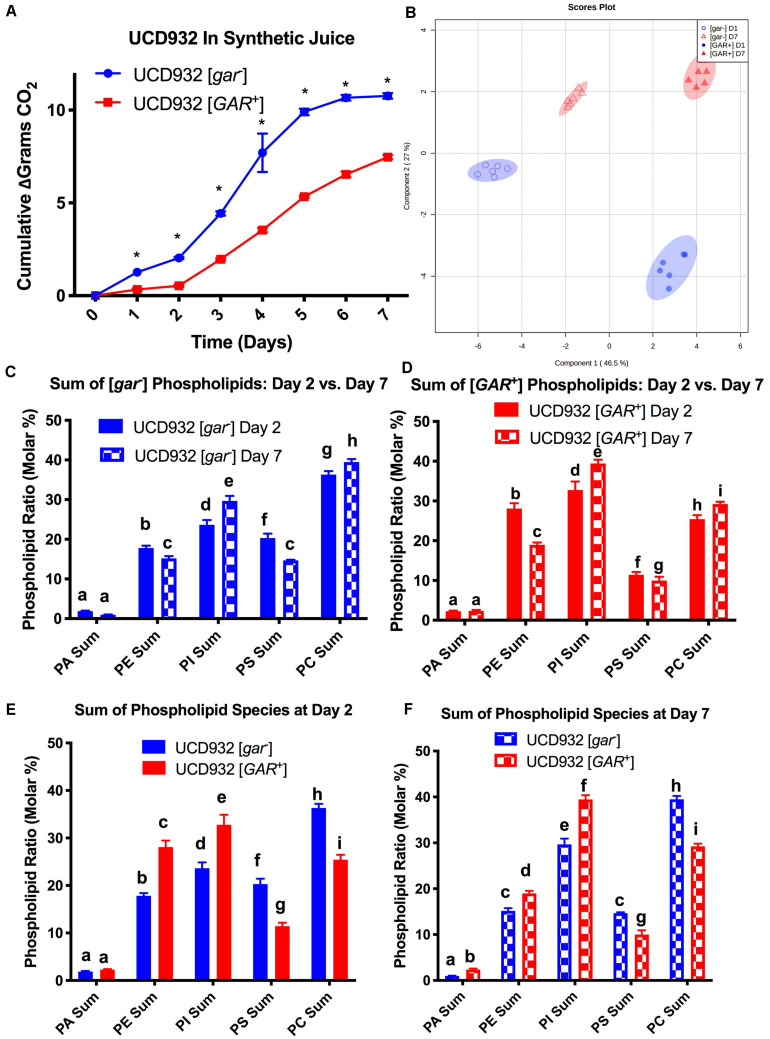
Segregation of Phospholipid Headgroup Species by Prion and Fermentation Progression. **(A)** Progression of fermentation measured by loss of carbon dioxide (in grams) of triplicate UCD932 [*gar*^–^] and [*GAR*^+^] fermentations in synthetic juice. The asterisks denote significantly different values between conditions as determined by paired sample *t*-test (*p*≤ 0.05). **(B)** PLS-DA plot of UCD932 [*gar*^–^] and [*GAR*^+^] phospholipids on day 2 (early) and day 7 (late). **(C)** Summation of the UCD932 [*gar*^–^] phospholipid head groups (total mol %) and comparison of days 2 and 7. **(D)** Summation of the UCD932 [*GAR*^+^] phospholipid head groups (total mol %) and comparison of days 2 and 7. **(E)** Summation of the UCD932 [*gar*^–^] and [*GAR*^+^] phospholipid head groups (total mol %) on day 2. **(F)** Summation of the UCD932 [*gar*^–^] and [*GAR*^+^] phospholipid head groups (total mol %) on day 7. Different letters above the bars denote significant differences among the treatments as determined by Fisher’s least significant difference test (*p* ≤ 0.05).

Phospholipid composition changes over the course of fermentation as cells enter non-proliferative states and adapt to increasing ethanol concentrations (Henderson and Block, 2014). The correlation between yeast plasma membrane composition and fermentation capacity has been established (Henderson et al., 2013a,b). Given the observed differences in fermentation capacity of [*gar*^–^] and [*GAR*^+^] cells, we assessed phospholipid composition of these two cell types during fermentation. Variance in phospholipid headgroup and fatty acid species were compared. Initially, triplicate fermentations were conducted with UCD932 [*gar*^–^] to determine if this yeast strain displayed the typical plasma membrane lipid profiles previously described for yeast during a typical fermentation and compared to the prion-induced [*GAR*^+^] state. A synthetic juice medium was used, for fermentations, cells were sampled for lipid analysis at day 2 (early) and day 7 (late) (Figure 4A). As expected, the WT [*gar*^–^] strain displayed typical fermentation kinetics, while the [*GAR*^+^] strain exhibited sluggish growth and fermentation kinetics (Figure 4A).

The significance of phospholipid headgroup differences between the two physiological states was determined by *t*-test and ANOVA. The composition of phospholipid species in [*gar-*] and [*GAR*^+^] conditions was visualized by partial least squared discriminant analysis (PLS-DA), with PC1 and PC2 capturing 55.7 and 25.5% of the variability, respectively (Figure 4B). By this analysis, the [*gar*^–^] and [*GAR*^+^] conditions segregate from each other, as expected, as do the sampling days clearly showing that the prion states differ in membrane composition at both time points (Figure 4B).

The 2- and 7-days time points were chosen to facilitate comparison to previously published work correlating fermentation capacity to plasma membrane composition (Henderson et al., 2013a,b). In general, the phospholipid headgroup composition of [*gar*^–^] cells was characterized by a relatively high molar ratio of phosphatidylcholine (PC) phosphatidylinositol (PI) and phosphotidylserine (PS) lipid species, in that order (Figure 4C), which have been shown to be predictive of successful fermentations and increased ethanol tolerance (Mishra and Prasad, 1988; Henderson et al., 2013b). As the [*gar*^–^] fermentations progressed from days 2 to 7, the molar ratios of phosphatidylethanolamine (PE) and PS decreased while PC increased and phosphatidylinositol (PI) increased significantly (Figure 4C). Increase in membrane PI concentration is associated with stationary phase and the depletion of sugar, indicative of the [*gar*^–^] strains nearing the end of fermentation (Mishra and Prasad, 1988; Henderson et al., 2013a). Thus, the observed changes in lipid profile for UCD932 [*gar*^–^] are typical for what is seen in a fermentation progressing normally.

The lipid profile of [*GAR*^+^] cells in synthetic juice was evaluated at the same timepoints (Figure 4D). In contrast to the [*gar*^–^] condition, the phospholipid composition of [*GAR*^+^] cells was characterized by a relatively high molar ratio of phosphatidylinositol (PI) and phosphatidylethanolamine (PE) headgroups. Increased concentrations of PI in the cell membrane has been shown to be predictive of problematic fermentations, while increased PE is associated with low temperature fermentations (Henderson et al., 2013a,b). As the [*GAR*^+^] fermentations progressed from days 2 to 7, PS and PE decreased significantly, while PC and PI increased significantly (Figure 4D). Early in fermentation [*gar*^–^] cells displayed a higher relative concentration of PC and PS species, while [*GAR*^+^] cells have a higher relative concentration of PI and PE despite beginning rapid sugar utilization. By day 7 of fermentation, [*gar*^–^] cells have finished fermentation, and display a molar ratio of PS and PE more similar to the [*GAR*^+^] cells still in mid-fermentation. The molar ratio of PI and PC remain relatively similar between [*gar*^–^] and [*GAR*^+^] conditions (Figures 4E,F). The high molar ratio of PI in [*GAR*^+^] cells suggest that this population is already in a sugar-depleted mode even in early and mid-fermentation. The prion state appears to be correlated with plasma membrane phospholipid composition associated with reduced fermentation capacity. Consistent with this observation, the [*GAR*^+^] fermentations did not start rapid sugar utilization until between days 2 and 3 in contrast to the isogenic [*gar-*] strain. The phospholipid profiles of [*GAR*^+^] cells are similar to those found under conditions of fermentative stress (Henderson et al., 2013a,b).

### Plasma Membrane Phospholipid Fatty Acid Composition Also Differentiates [*gar*^–^] and [*GAR*^+^] Cells

In addition to assessment of differences in content of phospholipid headgroup species, the composition of the fatty acid tails attached to the headgroups was also evaluated. The major fatty acids present at the *sn-1* and *sn-2* position on the glycerol moiety of phospholipids present in *S. cerevisiae* are oleic acid (C_18:1_) and palmitoleic acid (C_16:1_), and to a lesser extent palmitic acid (C_16:0_) and stearic acid (C_18:0_) (Gaspar et al., 2007; van Meer et al., 2008; Henderson and Block, 2014). In addition to phospholipid headgroup composition, the relative concentration of these fatty acid chains is correlated with fermentation rate, biomass accumulation, and ethanol tolerance (Henderson and Block, 2014).

**FIGURE 5:**
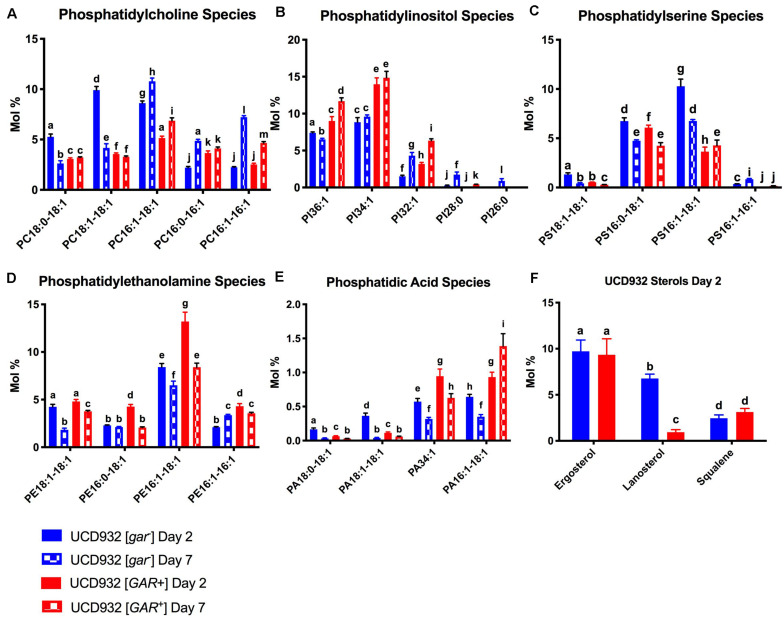
Segregation of Phospholipid Fatty Acid Species and Sterols During Fermentation.**(A)** Comparison of mol % of phosphatidylcholine (PC) species between UCD932 [*gar*^–^] and [*GAR*^+^] on days 2 and 7. **(B)** Comparison of mol % of phosphatidylinositol (PI) species between UCD932 [*gar*^–^] and [*GAR*^+^] on days 2 and 7. **(C)** Comparison of mol % of phosphatidylserine (PS) species between UCD932 [*gar*^–^] and [*GAR*^+^] on days 2 and 7. **(D)** Comparison of mol % of phosphatidylethanolamine (PE) species between UCD932 [*gar*^–^] and [*GAR*^+^] on days 2 and 7. **(E)** Comparison of mol % of phosphatidic acid (PA) species between UCD932 [*gar*^–^] and [*GAR*^+^] on days 2 and 7. **(F)** Comparison of mol % of UCD932 [*gar*^–^] and [*GAR*^+^] sterol composition on day 2. Different letters above the bars denote significant differences among the treatments as determined by Fisher’s least significant difference test (*p* ≤ 0.05).

The [*gar*^–^] cells exhibit a relatively high mol % of PC16:1–18:1, PC18:1–18:1, and PC18:0–18:1 on day 2 of fermentation. While PC16:1–18:1 remains elevated on day 7, PC16:1–16:1, and PC16:0–16:1 have increased relative to day 2 (Figure 5A). In contrast the [*GAR*^+^] cells exhibited a lower overall molar ratio of these PC species regardless of fermentation phase, and smaller relative changes in the molar ratio of PC species between days 2 and 7 (Figure 5A).

**FIGURE 6:**
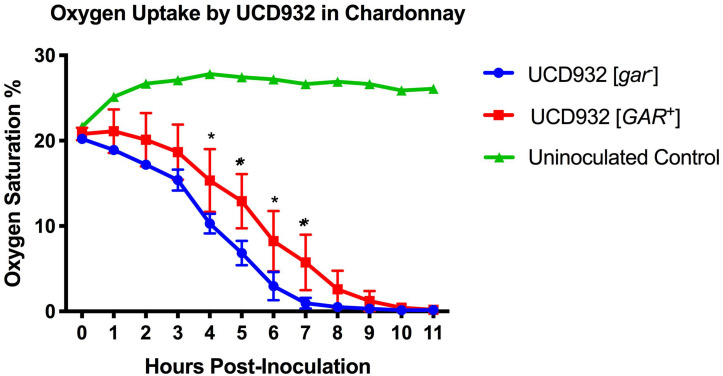
Oxygen Uptake During Fermentation. Uptake kinetics of dissolved oxygen by UCD932 [*gar*^–^] and [*GAR*^+^] in sterile filtered Chardonnay as measured with Pst3 iron oxide dots. The error bars represent the standard error of the mean in dissolved oxygen measurements of triplicate flasks at each time point. The asterisks denote significantly different values between conditions as determined by paired sample *t*-test (*p* ≤ 0.05).

The [*GAR*^+^] cells generally have a higher molar ratio of PI when compared to [*gar*^–^] cells, particularly of the mol % of PI32:1, PI34:1, and PI36:1 species at days 2 and 7 despite being at an earlier phase of fermentation (Figure 6B). Fermentations of [*gar*^–^] cells entering stationary phase by day 7 showed an increased mol% of the shorter chain PI species such as PI:28:0 and PI32:1, but generally still had lower relative concentrations of the major PI species than [*GAR*^+^] cells in mid-fermentation (Figure 5B).

The [*gar*^–^] cells initially had more PS but both conditions display a decrease in PS between sampling points, just more dramatically with [*gar*^–^] cells. Both conditions exhibit similar mol % of PS16:0–18:1 at day 2, and a similar relative drop by day 7 (Figure 5C). The mol% of PS16:1–18:1 is much higher on day 2 for [*gar*^–^] than [*GAR*^+^] but drops by day 7, while the mol % increases slightly for [*GAR*^+^] (Figure 5C). Similar to PI and PC, the shorter chain lengths appear to be increasing slightly between day 2 and 7 for the [*gar*^–^] cells while the [*GAR*^+^] relative concentrations remain more constant between time points.

The [*GAR*^+^] cells initially had a slightly higher molar ratios of PE, this difference was increased by day 7. [*GAR*^+^] cells had a high mol % of PE16:1–16:1, PE16:1–18:1, and PE16:0–18:1 compared with [*gar*^–^] on day 2, but by day 7 PE16:1–16:1 had increased for [*gar*^–^] and decreased for [*GAR*^+^], PE16:1–18:1 had decreased for both [*gar*^–^] and [*GAR*^+^], and PE16:0–18:1 had decreased in [*GAR*^+^] till equivalent with [*gar*^–^] (Figure 5D).

Although PA is a proportionally small part of the overall molar composition, it is important as a precursor to other phospholipids and as a signaling molecule (Carman and Henry, 2007; Shin and Loewen, 2011). At day 2 when [*gar*^–^] cells were early-mid fermentation and [*GAR*^+^] cells were still rapidly utilizing sugar, [*gar*^–^] had a higher mol % of PA18:1–18:1 and PA18:0–18:1 but [*GAR*^+^] had a higher mol % of PA16:1–18:1 and PA:34:1 (Figure 5E). The [*gar*^–^] cells entering stationary phase on day 7 showed a drop in almost all PA species, while [*GAR*^+^] cells still in mid-fermentation showed a distinct rise in the mol % of PA16:1–18:1 (Figure 5E).

Sterol composition was also observed on day 2 of fermentation. Relative molar ratios of ergosterol and squalene were similar, but the molar ratio of lanosterol was higher in [*gar*^–^] than [*GAR*^+^] cells (Figure 5F). Cumulatively, all of these differences in plasma membrane composition between [*gar*^–^] and [*GAR*^+^] cells are indicative of reduced fermentation capacity observed in prion-induced cells. Our analysis showed that ergosterol was the major species present in both [*gar*^–^] and [*GAR*^+^] cells, but that the precursor lanosterol was significantly lower in the [*GAR*^+^] early fermentation condition. Given the role of lanosterol as a biosynthetic intermediate between squalene and ergosterol, the accumulation of lanosterol could be correlated to the slower fermentation rate of [*GAR*^+^] cells and reduced uptake of oxygen (Fornairon-Bonnefond et al., 2002).

### [*GAR*^+^] Cells Display a Reduced Rate of Oxygen Uptake

The differences in membrane composition suggested that membrane functionality may differ between [*gar*^–^] vs. [*GAR*^+^] cells. Depletion of oxygen during fermentation is not only important for the dominance of *Saccharomyces* over other microbes present, but is also crucial for the synthesis of fatty acids and sterols (Rosenfeld et al., 2003). The ability to deplete oxygen from the environment is dependent upon the ability to create an electrochemical gradient across the plasma membrane (Boulton et al., 1996). Given the observed differences in phospholipid composition and potential changes in plasma membrane functionality between [*gar*^–^] and [*GAR*^+^] cells, we hypothesized that there would be differences in O_2_ uptake during fermentation. Indeed, when concentrations of dissolved oxygen were tracked in sterile filtered Chardonnay, it was observed that UCD932 [*GAR*^+^] cells consumed O_2_ less efficiently than [*gar*^–^] cells (Figure 6). Differences in oxygen consumption are most pronounced by hour 4 post-inoculation, indicative of differences in growth rate and oxygen consumption abilities. While these differences may appear relatively minor for small-scale isogenic strain lab conditions, juice used in winemaking would have a full complement of wild organisms. Any reduction in O_2_ uptake could markedly affect the competitiveness of *Saccharomyces* in fermentation by allowing increased growth and metabolic activity of microbial competitors (Boulton et al., 1996). Molecular O_2_ in solution could allow for further proliferation of bacteria that can inhibit fermentation and potentially induce more yeast to adopt the [*GAR*^+^] prion state (Jarosz et al., 2014a).

## Discussion

Induction of [*GAR*^+^] enables a yeast population to respond dynamically and transiently to bacterial competition and inhibitor formation in mixed microbial communities. The organic acids produced by bacteria, especially acetic acid (Ramakrishnan et al., 2016), are detrimental to yeast and, depending on the strain history/environmental niche, a portion of a population will induce the [*GAR*^+^] prion due to the disruption of cellular homeostasis (Ullah et al., 2013; Garcia et al., 2016; Ramakrishnan et al., 2016). By induction of the prion state yeast benefit by increasing cell survivability albeit at the cost of downshifting their dominant fermentative behavior. Bacteria benefit from this shift in the yeast population as available oxygen allows them to better compete with the yeast for nutrients, while reduced ethanol allows them to remain metabolically active for more of the fermentation (Ramakrishnan et al., 2016).

Previous work characterized a major consequence of [*GAR*^+^] prion induction as the reduction of expression of a major hexose transporter, *HXT3* (Brown and Lindquist, 2009). The loss of fermentative capacity and glucose repression was thought to be due to the decreased level of sugar transport. This work focused on evaluation of expression profiles in laboratory strains at the transition from aerobic growth on glucose to growth on ethanol, termed the diauxic shift, a transition that does not occur during grape juice fermentation. The observation that wine strains display high expression of the [*GAR*^+^] prion suggests that there is some advantage to prion induction. The *HXT3* transporter is the major transporter expressed during fermentation and if present as the sole *HXT3* gene leads to near normal fermentation profiles (Reifenberger et al., 1997). However, the complete loss of Hxt3 does not prevent fermentation as other transporters become more highly expressed (Perez et al., 2005; Karpel et al., 2008). Also, during non-proliferative fermentation the correlation between expression level and protein level is less robust than during active growth. Indeed, there was a reduction in Hxt3 protein observed in the plasma membrane of UCD932 [*GAR*^+^]. However, the fact that induction of the prion in an *hxt3* null strain resulted in further loss of fermentative capacity suggests that either expression of other *HXT* genes is also impacted, or that other changes in the cells had occurred.

The changes observed in [*GAR*^+^] as compared to [*gar*^–^] cells suggest a boarder adaptation of the cells to stressful conditions than would occur from simple reduction of fermentative capacity. The changes in cell morphology suggest that prion state induction remodels the cell surface. Differences in plasma membrane phospholipid composition are consistent with the fermentation behavior of prion induced and non-induced strains. The composition of phospholipids in the yeast membrane can affect growth rate, fermentation capacity, protein structure/activity, alcohol production/tolerance, along with intracellular signaling and homeostasis (van Meer et al., 2008; Henderson and Block, 2014).

Additionally, the composition of different phospholipid headgroups can be predictive of fermentative performance and indicative of physiological differences induced by the environment (Henderson et al., 2013a). The biophysical properties of the different phospholipid headgroups are correlated with different functions (Bigay and Antonny, 2012). Phosphatidylcholine (PC), has been implicated as a reservoir for cell signaling molecules (de Kroon, 2007). Phosphatidylinositol (PI),is involved in cell growth and alcohol tolerance, along with being a precursor to various inositol species important for cell signaling (Gardocki et al., 2005; Strahl and Thorner, 2007). Phosphatidylserine (PS),is involved with alcohol tolerance and cell signaling (Vance and Steenbergen, 2005; Kay et al., 2012). Phosphatidylethanolamine (PE), positively regulates longevity and plays a role in cell signaling (Rockenfeller et al., 2015). Finally, Phosphatidic acid (PA), serves a precursor to all other phospholipids and is highly sensitive to changes in pH and is important for intracellular sensing (Young et al., 2010; Shin and Loewen, 2011).

With respect to plasma membrane lipid composition the most obvious and robust difference between [*gar*^–^] and [*GAR*^+^] cells is the relative concentrations of PC and PI. High relative concentrations of PC as seen in the wild type [*gar*^–^] state have been correlated with increased cell density and successful fermentations (Henderson et al., 2013a). In contrast, the high relative concentrations of PI observed in the [*GAR*^+^] state have been correlated with reduced cell density and problematic fermentations (Henderson et al., 2013a). Generally, as cells progress in fermentation and transition from exponential to stationary phase, the molar ratio of PI increases due to nitrogen depletion (Henderson et al., 2013b). Although PI did increase in [*gar*^–^] cells from days 2 to 7, [*GAR*^+^] cells started out with a high relative concentration of PI and all major species of this phospholipid increased from days 2 to 7. PI is also precursor to phosphoinosides, important in cell signaling and regulatory networks (Strahl and Thorner, 2007). Increased concentrations of PI in [*GAR*^+^] could be indicative of problems with phosphoinositide turnover/synthesis and the disruption of metabolism, growth, and sugar utilization (Henderson et al., 2013b). The ratio of PI to PC in [*GAR*^+^] cells, implies that they are in an alternate physiological state even during exponential growth.

The other clear difference between the wild type and prion conditions was the high relative concentrations of PS and lower relative concentration of PE in [*gar*^–^] cells compared with lower concentration of PS and higher concentration of PE in [*GAR*^+^] cells. Both PS and PE levels decreased as the fermentations progressed. Increased relative concentrations of PS have been shown to be associated with ethanol tolerance (Mishra and Prasad, 1988; Zinser et al., 1991). Due to the biophysical properties of PS, it is essential for membrane curvature, budding, and transport vesicle formation (Fuller et al., 2003; Xu et al., 2013). Lower relative concentrations of PS could contribute not only to reduced alcohol tolerance in [*GAR*^+^] cells but also to the observed incomplete bud separation/clumping phenotypes (Kay et al., 2012). Like PS, PE induces (negative) membrane curvature and is located primarily on the cytosolic facing leaflet. PE plays distinct roles in regulating autophagy and longevity of cells; increased relative concentrations of PE have been shown to increase autophagic flux thus extending the chronological life span of yeast cells (Rockenfeller et al., 2015). Just as the PC:PI molar ratio is predictive of fermentative capacity, we hypothesize that the PS:PE molar ratio may be a fundamental indicator of [*gar*^–^] vs. [*GAR*^+^] protein function, membrane physiology, and metabolism. Greater longevity after arrest of sugar consumption has been observed in [*GAR*^+^] cells (Walker et al., 2016) and this correlates with higher relative PE concentration (Rockenfeller et al., 2015).

Early and late in fermentation PA remains relatively elevated in [*GAR*^+^] cells. The role of PA in pH sensing and the ability of bacterially-produced acids to induce the prion state suggest that prion induction may be more related to proton stress and stressors in the environment than as a strategy to simply metabolize alternative carbon sources in the presence of glucose.

Ethanol tolerance in yeast is generally a function of the permeability and biophysical properties of the phospholipid bilayer (Schiavone et al., 2016). As fermentation progress, ethanol concentrations increase, fluidizing and thinning the plasma membrane by up to 30%. As a result, passive proton flux increases and can impact integral protein structure and function (Henderson and Block, 2014). Yeast mitigate the thinning effects of increasing ethanol concentrations by lengthening fatty acid tails and increasing the degree of saturation to help solidify the membrane in the presence of ethanol (Henderson and Block, 2014). Sterols in yeast have long been understood to aid ethanol tolerance by helping to maintain lipid raft domains and mitigate the thinning effects of ethanol (Stanley et al., 2010). However, the actual data on the effect of sterols in the membrane is somewhat mixed, being highly dependent on strain and growth condition (Henderson and Block, 2014).

Rapid depletion of molecular oxygen is a factor enabling *Saccharomyces* to establish dominance in wine fermentations, since most microbial competitors are dependent on oxygen for their metabolism and adaptation to growth in juice (Boulton et al., 1996). The uptake of molecular O_2_ is a function of membrane potential, established via metabolic flux and the ATPase activity of Pma1 (Toulmay and Schneiter, 2007). The kinetics of O_2_ uptake in [*GAR*^+^] was slower in sterile filtered media than for the wildtype strain. Reduced O_2_ uptake could in turn affect the composition of lipids/sterols, fermentation kinetics, and metabolic flux (Rosenfeld et al., 2003).

The induction of [*GAR*^+^] has been attributed to the association between Pma1 and Std1 and that association has been well characterized (Brown and Lindquist, 2009). Std1 and Pma1 are required for the establishment of the prion state but Std1 is not required for maintenance (Brown and Lindquist, 2009). Std1 is a known transcriptional regulator of multiple transcriptional processes (Ganster et al., 1993; Tillman et al., 1995; Simpson-Lavy and Kupiec, 2018). The binding of Std1 to Pma1 may stabilize not only its role as a regulator of hexose transporter expression via Rgt1 but also of glucose repression via Snf1. Finally, Std1 is known to self-aggregate and form a non-amyloid “puncta” structure inactivating its role in transcription (Simpson-Lavy and Kupiec, 2018). Binding to Pma1 would prevent this inactivation and enable expression and repression of Std1- regulated genes to occur. Transient overexpression of Std1 leads to prion induction further supporting the view that regulatory processes dependent upon free Std1 are involved in the suite of physiological changes observed (Brown and Lindquist, 2009).

Our discovery that acetate specifically induces the prion state in wine strain UCD932 is also an intriguing observation (Ramakrishnan et al., 2016).). Acetate functions as a stress signal within the cell, not at the cell surface (Simpson-Lavy and Kupiec, 2019a,b). Acetate resistant membranes are higher in PI content (Guo et al., 2018), consistent with what is observed for [*GAR*^+^] cells. In general, late in fermentation cells become more impermeable to H^+^ ions (Viana et al., 2012) so the lipid changes observed in [*GAR*^+^] cells may suggest and early adaptation to the presence of proton stress in the environment.

The identification of the [*GAR*^+^] prion state suggests that tolerance to acetic acid can become inherited in the absence of continued stress of acetic acid. Since resistance to acetic acid leads to cell surface changes that decrease permeability of this acid and since it is toxic internally and signals as a stressor internally, blocking of its uptake would be predicted to lead to a reversal of tolerance as acetate dissipates from the cell. A heritable memory of the presence of acetic acid in the environment would lead to enhanced cell survival, particularly in mixed-culture environments. The nature of that heritable memory remains elusive. It is unclear how the specific changes in membrane composition observed are inherited by progeny cells.

## Conclusion

Comparison of the impact of induction of the [*GAR*^+^] prion in wildtype and *hxt3Δ* strains of the vineyard isolate, UCD932, revealed that factors in addition to decreased expression of the hexose transporter underlie the reduced fermentative capacity associated with the prion. Colony, cell and plasma membrane morphology were found to differ between the two cellular states suggesting remodeling of the cell surface. In addition, plasma membrane phospholipid content varied in ways consistent with differences in fermentation capacity. Finally, membrane functionality, as measured by oxygen uptake also differed. We propose that, in the wild, prion establishment leads to a retooling of cellular metabolism and plasma membrane functionality. These changes serve as a dynamic protective mechanism to avoid cell damage under conditions where normal metabolic processes would be deleterious. These findings raise a fundamental question of the nature of the heritable unit associated with [*GAR*^+^] establishment. Further studies should elucidate the nature of inheritance of the [*GAR*^+^] phenotype.

## Data Availability Statement

The raw data supporting the conclusions of this article will be made available by the authors, without undue reservation, to any qualified researcher. The raw data for the lipidomics analysis is available as a .cvs file in Supplementary Material.

## Author Contributions

GW, LB, DB, and CH planned the studies. GW conducted the research with input from PL. CH conducted the lipidomic analysis. GW wrote the first draft. LB and GW wrote the final draft. All authors contributed to review and editing of the final draft.

## Conflict of Interest

The authors declare that the research was conducted in the absence of any commercial or financial relationships that could be construed as a potential conflict of interest.

## Supplementary Material

FIGURE S1 Data in this figure are the mean and standard deviation of the derivative of the rate of CO_2_ loss (in grams) per day of six biological replicates for each condition presented. A pair-wise *t*-test was performed to determine when the Brix between conditions was significantly different (*p* ≤ 0.05), asterisks have been added to denote significance. **(A)** The rate of fermentation of UCD932 [*gar*^–^] is compared to the rate of UCD932 [*gar*^–^] *hxt3Δ* in synthetic juice, weight loss measured as CO_2_ evolution to track fermentation progress. **(B)** The rate of fermentation of UCD932 [*GAR*^+^] is compared to UCD932 [*GAR*^+^] *hxt3Δ* in synthetic juice **(C)** The rate of fermentation of UCD932 [*gar*^–^] is compared to UCD932 [*GAR*^+^] in synthetic juice. **(D)** The rate of fermentation of all strains is compared together in synthetic juice.

FIGURE S2 Additional fields of view for the pictures presented in Figure 3. **(A)** UCCT 1405 Pma1:mNeon Exponential Phase. **(B)** UCD932 Hxt3-GFP Exponential Phase **(C)** UCD932 Hxt3-GFP Stationary Phase.
