# Supplementary figures and images for: Downshifting Yeast Dominance: Cell Physiology and Phospholipid Composition Are Altered With Establishment of the [GAR+] Prion in Saccharomyces cerevisiae
Source: Front Microbiol. 2020 Aug 25;11:2011. doi: 10.3389/fmicb.2020.02011 (PMC7477300; doi:10.3389/fmicb.2020.02011)

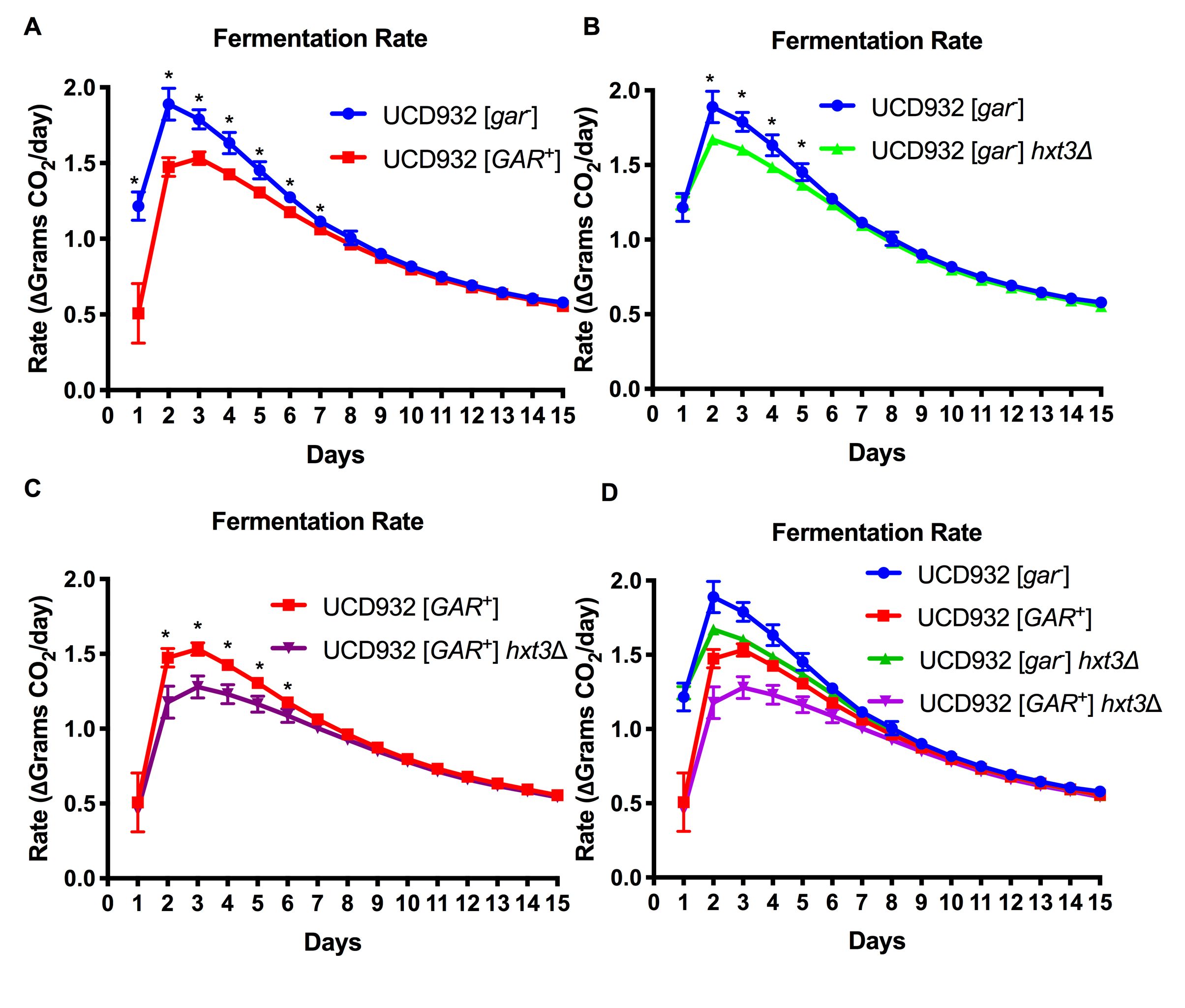

Supplement: FIGURE S1 — Data in this figure are the mean and standard deviation of the derivative of the rate of CO2 loss (in grams) per day of six biological replicates for each condition presented. A pair-wise t-test was performed to determine when the Brix between conditions was significantly different (p ≤ 0.05), asterisks have been added to denote significance. (A) The rate of fermentation of UCD932 [gar–] is compared to the rate of UCD932 [gar–] hxt3Δ in synthetic juice, weight loss measured as CO2 evolution to track fermentation progress. (B) The rate of fermentation of UCD932 [GAR+] is compared to UCD932 [GAR+] hxt3Δ in synthetic juice (C) The rate of fermentation of UCD932 [gar–] is compared to UCD932 [GAR+] in synthetic juice. (D) The rate of fermentation of all strains is compared together in synthetic juice. [file Image_1.JPEG]

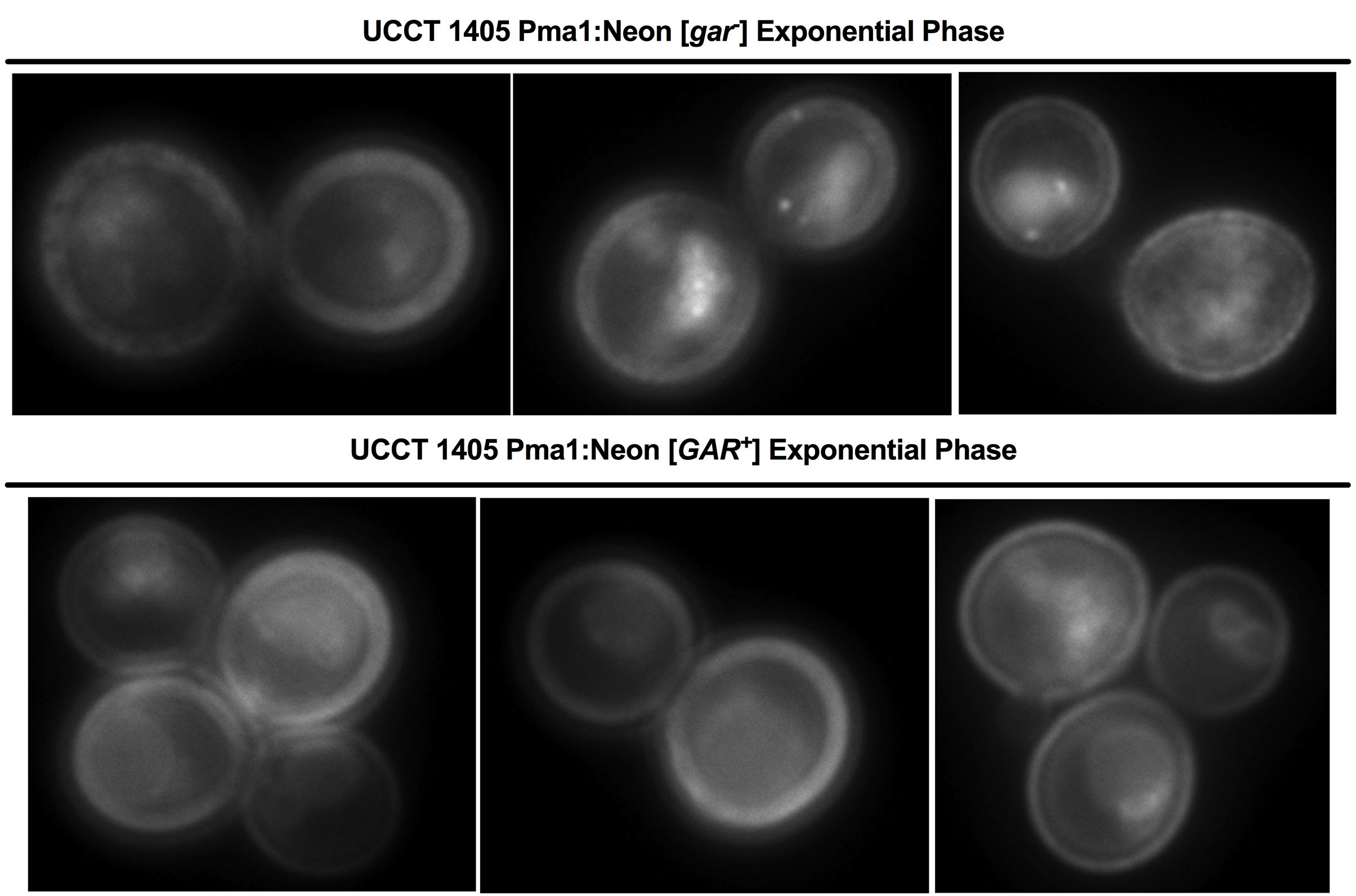

Supplement: FIGURE S2 — Additional fields of view for the pictures presented in Figure 3. (A) UCCT 1405 Pma1:mNeon Exponential Phase. (B) UCD932 Hxt3-GFP Exponential Phase (C) UCD932 Hxt3-GFP Stationary Phase. [file Image_2.JPEG]

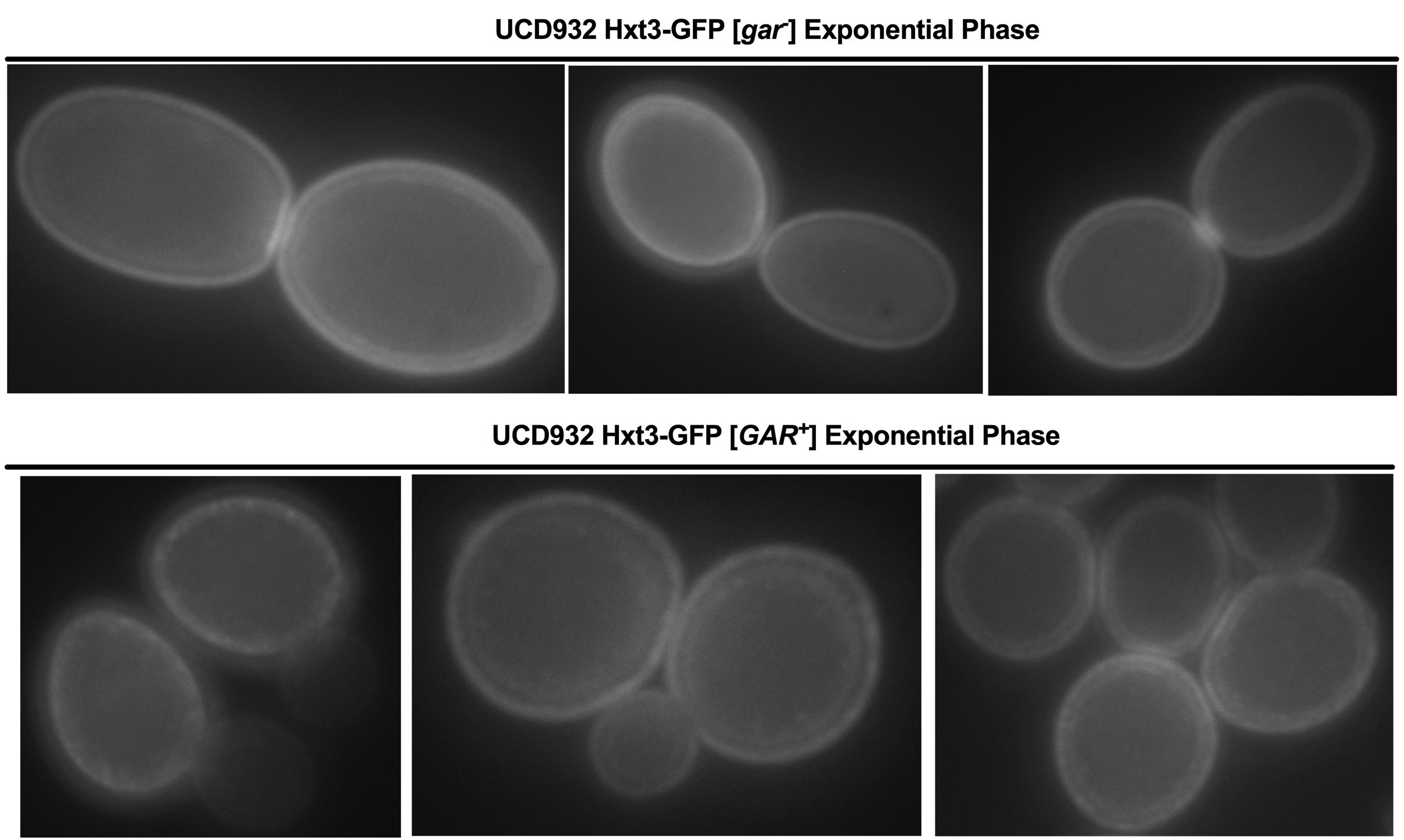

Supplement: Supplementary file 3 [file Image_3.JPEG]

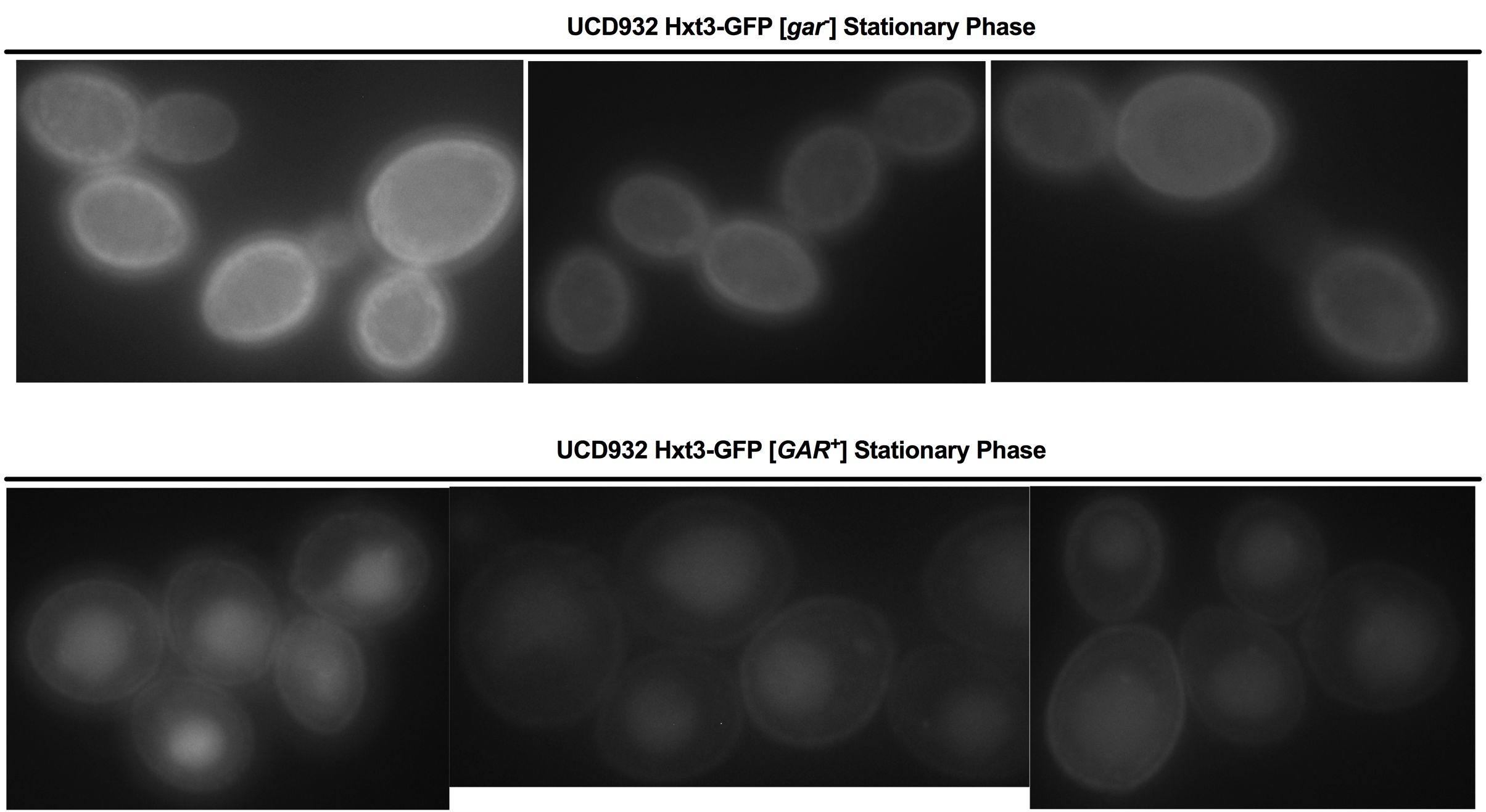

Supplement: Supplementary file 4 [file Image_4.JPEG]
